# Comparative Analysis of Proteomic Characteristics in Seminal Plasma Between Horses and Donkeys

**DOI:** 10.3390/ani15111532

**Published:** 2025-05-23

**Authors:** Xin Wen, Hong Ren, Qianqian He, Minna Yi, Tseweendolmaa Ulaangerel, Gerelchimeg Bou

**Affiliations:** Inner Mongolia Key Laboratory of Equine Science Research and Technology Innovation, Inner Mongolia Agricultural University, Hohhot 010018, China; wenxin618@imau.edu.cn (X.W.); renhong1980@126.com (H.R.); qianqianhe202309@163.com (Q.H.); yiminna2020@163.com (M.Y.); cewendclma@126.com (T.U.)

**Keywords:** seminal plasma, proteomic profile, 4D-DIA, horse, donkey, equid, pregnancy rate

## Abstract

Horses and donkeys, as integral members of the equine family, exhibit distinct reproductive capabilities and characteristics. Seminal plasma, the fluid component of semen, contains a variety of proteins that play critical roles in sperm function and fertility. This study aimed to systematically compare the protein profiles in the seminal plasma of horses and donkeys, thereby elucidating the molecular differences between these two species. The study utilized 4D-DIA proteomics technology to analyze seminal plasma from horses and donkeys and further validated key proteins through Western blot. Our findings revealed significant variations in seminal plasma protein composition between horses and donkeys. We identified 2380 and 2385 proteins in the seminal plasma of horses and donkeys. Among these proteins, 59 are solely present in the seminal plasma of horses, and 64 uniquely exsit in that of donkeys, respectively. These insights enhance our understanding of the biological mechanisms underlying the reproductive distinctions between these equine species. Moreover, the identified species specific proteins may be essential for thier sperm quality and function, which holds practical value for breeding programs and investigations.

## Simple Summary

This study conducted a comparative proteomic analysis of the seminal plasma of horses and donkeys, identifying and comparing the types, abundances, and functional differences of proteins in their seminal plasma. It revealed the potential molecular mechanisms underlying the different reproductive characteristics of horses and donkeys, providing a basis for understanding equid reproductive biology.

## 1. Introduction

Horses and donkeys, as key members of the equine family, have played pivotal roles in human history, serving not only as working animals and companions but also as essential contributors to agriculture and the equestrian industry [1,2,3]. Their distinct reproductive traits, including variations in fertility rates, semen quality, and interspecies reproductive barriers, have long fascinated both researchers and practitioners [4,5,6]. However, despite their economic and biological significance, comprehensive comparative analyses of the proteomic profiles in the seminal plasma of these two species remain scarce. This knowledge gap impedes the development of targeted strategies aimed at enhancing reproductive efficiency, optimizing breeding programs, and addressing interspecies reproductive challenges.

Seminal plasma serves as a vital secretion within the reproductive tract of male animals and contains seminal plasma proteins that are secreted mainly by accessory glands such as the prostate and seminal vesicles [7,8]. These proteins not only offer essential nutrients and protection for sperm but also play key roles in sperm generation, maturation, activation, movement, and fertilization processes [9,10]. This directly influences the reproductive potential of male animals. Specific seminal plasma proteins have been linked to human fertility, as well as that of mice, cows, sheep, and pigs [11,12,13,14,15]. Four-dimensional data-independent acquisition (4D-DIA) protein sequencing technology represents a proteomics analysis approach grounded in mass spectrometry, integrating ion mobility and the synchronous accumulation of charged particles by velocity filtering (PASEF) technologies to enable efficient and high-resolution analysis of intricate protein samples [16,17]. This innovative method introduces the collision cross-section (CCS) of peptide segments as the fourth dimension, alongside traditional three-dimensional separation parameters (retention time, mass-to-charge ratio, and ionic strength), thereby achieving the four-dimensional separation and detection of protein samples [17]. This four-dimensional separation mode not only enhances the precision and reliability of protein identification but also markedly reduces sample complexity and background noise, facilitating the detection of low-abundance proteins [18]. Recent research has demonstrated that 4D-DIA revealed protein characteristics to distinguish malignant pleural effusion from benign pleural effusion [19]. In the field of reproductive biology, the 4D-DIA method elucidates the dynamics of protein composition and changes in sperm motility by analyzing 14 candidate proteins that may influence the cryopreservation capability of goat sperm [20]. In conclusion, 4D-DIA protein sequencing technology appears to be the most potent tool currently available for identifying protein characteristics.

Therefore, we hypothesize that significant differences exist in the proteomic profiles of seminal plasma between horses and donkeys, reflecting their distinct reproductive physiologies and evolutionary adaptations. This study aims to systematically characterize and compare the proteomic profiles of equine and donkey seminal plasma, identifying species-specific proteins that may underlie differences in fertility and semen quality. The insights derived from this analysis are expected to enhance our understanding of the molecular mechanisms governing reproduction in equids and support the development of targeted biomarkers for semen evaluation. Furthermore, this research represents a critical contribution to advancing comparative proteomics within the equine family.

## 2. Materials and Methods

### 2.1. Experimental Design

**Figure 1:**
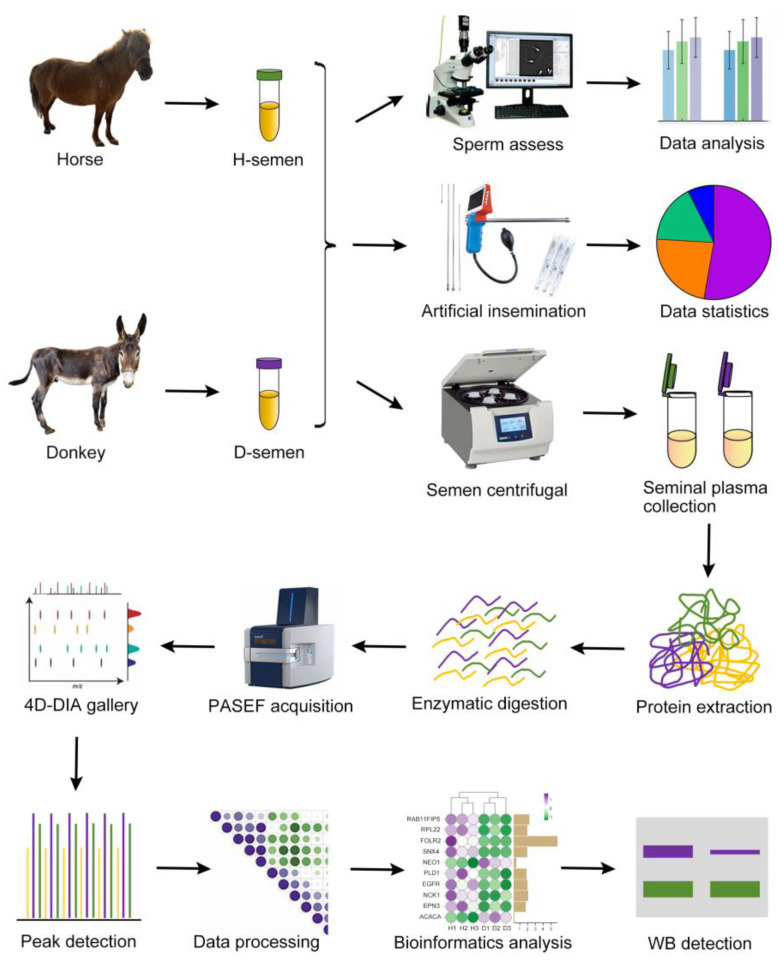
Schematic of the experimental design and workflow. Horse and donkey semen were collected, with a portion utilized for assessing sperm quality and artificial insemination, and the remainder was subjected to centrifugation to isolate seminal plasma for subsequent protein sequencing. The identification of key proteins was confirmed through Western blotting.

Semen was collected from three male horses and three male donkeys via artificial vaginal insemination. A portion of the semen was utilized for assessing sperm quality to gather statistical data on sperm morphology and motility. The other portion of the semen was used for artificial insemination, and the resulting pregnancy-related data were compiled. The remaining semen was centrifuged to obtain seminal plasma, which was then processed for protein extraction, enzymatic digestion, PASEF acquisition, spectral library search, peak identification, data analysis, and bioinformatics interpretation. The key proteins were subsequently verified through Western blotting experiments. A schematic illustration of the experimental design and workflow is presented in Figure 1.

### 2.2. Trial Animals

In this study, we worked with three male Mongolian horses and three male North China donkeys. The horses were 5 years old, on average (ranging from 4 to 6 years), and weighed between 280 and 350 kg, while the donkeys were also 5 years old, on average (ranging from 4 to 6 years), and weighed between 220 and 250 kg. All male animals were confirmed to be fertile through semen quality analysis and reproductive history checks. Additionally, 102 healthy mares and 76 jennies aged 4–8 years were included for artificial insemination experiments. These females all had a proven record of successful pregnancies. Sperm collection and testing, as well as seminal plasma collection and sequencing, took place during the breeding season from June to August 2023. Data on the number of artificially inseminated females were collected up to January 2025. Throughout the experiment, none of the animals received any medications, and comprehensive health checks confirmed that they were free of diseases. They were housed individually in comfortable stables at the Sport Horse Training Center of Inner Mongolia Agricultural University, where the temperature was kept between 18 and 25 °C, humidity at 40–60%, and an automatic ventilation system ensured fresh air circulation. Each animal received standard feed and water daily. All procedures followed strict guidelines and were approved by the Animal Protection Committee of Inner Mongolia Agricultural University.

### 2.3. Sperm Assessment

Five microliters of horse and donkey sperm samples (the sperm was diluted to a concentration of 5 × 10^7^/mL using the INRA-96^®^ (016441, IMV Technologies, Léger, France)) was dispensed into the Makler chamber (Sefi Medical Instruments, Haifa, Israel) of the CEROS II analyzer (Hamilton Thorne, Beverly, MA, USA). Sperm morphology (bent tail, coiled tail, DMR, distal droplet, and proximal droplet) and motility (percentages of static, progressive, motile, and slow) were assessed via an Axiolab A1 microscope (Olympus, Tokyo, Japan) according to predetermined parameters.

### 2.4. Artificial Insemination

From the onset of estrus to the detection of ovulation, daily transrectal ultrasound examinations were performed on mares and jennies. When a 35 mm follicle was identified in mares and a 30 mm follicle in jennies, estrous females were allocated to individual stalls for intraspecific artificial insemination. Artificial insemination was conducted every 48 h until ovulation occurred. Collected horse and donkey semen was diluted with appropriate diluents in proportion to ensure that the number of effective sperm introduced into the female reproductive tract ranged between 200 million and 500 million. Following standard artificial insemination protocols, the position of the cervix was determined by manual palpation. Subsequently, the artificial insemination catheter was carefully inserted into the uterine cavity and connected to a syringe for semen injection. After the semen was successfully deposited in the uterus, the catheter was withdrawn, and gentle massage of the cervix was performed to minimize the risk of semen backflow. The above description is based on Fanelli’s content [4]. Simultaneously, 45 to 60 days post-artificial insemination, the pregnancy status was confirmed using ultrasound examination and additional diagnostic methods.

### 2.5. Seminal Plasma Preparation

Following ejaculation, the semen sample was promptly centrifuged at 2000× *g* for 10 min to collect the supernatant. The supernatant was then subjected to a second centrifugation at 1000× *g* for 10 min to remove residual sperm and cellular debris. Subsequently, the supernatant was supplemented with 1% *v*/*v* proteinase inhibitor (P2714, Sigma, St. Louis, MO, USA) and stored at −80 °C until further analysis.

### 2.6. Protein Extraction and Digestion

The seminal plasma samples of horses and donkeys were combined with lysis buffer containing 8 M urea and a 1% proteinase inhibitor mixture. The mixture was sonicated using a high-intensity ultrasonic processor (Scientz, Ningbo, China) for 3 min. The mixture was centrifuged at 12,000× *g* for 10 min. Finally, the supernatant was collected, and the protein concentration was measured using a BCA reagent kit (Tiangen, Beijing, China). The prepared protein sample was added to a final concentration of 20% (*m*/*v*) TCA for protein precipitation. The mixture was thoroughly vortexed and then incubated for 2 h. The mixture was subsequently centrifuged at 4500× *g* for 5 min to collect the precipitate. To eliminate residual impurities, the precipitated protein was washed three times with precooled acetone and dried for 1 min at room temperature. The dried protein sample was subsequently reconstituted in 200 mM TEAB and sonicated. The sample was subjected to overnight trypsin (Promega, Madison, WI, USA) digestion while maintaining a trypsin-to-protein ratio of 1:50. Before digestion, the sample was treated with 5 mM dithiothreitol at 56 °C for 30 min and alkylated with 11 mM iodoacetamide in darkness for 15 min. Finally, peptide desalting was performed using a Strata × SPE column (Phenomenex, Torrance, CA, USA).

### 2.7. LC-MS/MS Detection

The trypsin peptide was dissolved in solvent A and loaded onto a custom-made reversed-phase column. The mobile phase consisted of solvent A (0.1% formic acid, 2% acetonitrile in water) and solvent B (0.1% formic acid, 90% acetonitrile in water). The peptide segments were separated and analyzed using an EASY nLC 1200 UPLC system (Thermo Fisher Scientific, Waltham, MA, USA) coupled with an Orbitrap Exploris 480 mass spectrometer. The Orbitrap detector scanned both precursors and fragments, while MS/MS scans were conducted on the basis of standard parameters.

### 2.8. Database Search

The search software utilized for the DIA mass spectrometry data was DIA-NN (v1.8.1), which employs the Libraryfree method for searching. The search parameters were as follows: uniprotkb_proteome_UP000002281_horse_20231115.fasta database (69,434 entries) for seminal plasma proteomic sequencing data in horses and uniprotkb_proteome_UP000694387_lv_20231115.fasta database (48,973 entries) for seminal plasma proteome sequencing data in donkeys. The selected options included using deep learning parameters to predict a library and employing MBR to generate a library from DIA data, followed by reanalyzing the DIA data via the generated library to obtain protein quantification, precursor ion identification, and protein-level FDR filtering with a 1% threshold.

### 2.9. Bioinformatics Analysis

Principal component analysis (PCA) was employed for dimensionality reduction and the visualization of multi-dimensional data, such as seminal plasma proteins. Specifically, the Scikit-learn library in Python (version 3.9.6) was utilized to perform PCA on standardized datasets, projecting the original data into the principal component space. The results were visualized using a two-dimensional scatter plot, where samples from different species (Mongolian horses and North China donkeys) were color-coded to clearly illustrate inter-group differences and the distribution patterns of the data. The eggNOG online database and KEGG Mapper online tool were utilized for KEGG analyses of the differentially expressed proteins (DEPs) identified between horse and donkey seminal plasma. Pathways with corrected *p* values < 0.05 were deemed to be significantly enriched.

### 2.10. PPI and Correlation Matrix

The protein–protein interaction (PPI) network was constructed using the STRING database (version 11.5) and visualized with Cytoscape (version 3.7.2). This study used the Pearson correlation coefficient to evaluate the linear relationship between variables and employed the corrplot package in R software (version 4.2.1) for calculation and visualization. The correlation matrix was generated by calculating the Pearson correlation coefficients between the seminal plasma proteins identified in horses and donkeys and their respective sperm vitality parameters. Prior to analysis, all continuous variables underwent normality testing using the Shapiro–Wilk test to confirm that the data met the assumptions required for Pearson correlation analysis. For non-normally distributed data, the Spearman rank correlation coefficient was applied. The significance level was set at *p* < 0.05, with extremely significant results defined as *p* < 0.01. The final results were displayed in a correlation heatmap, where the color intensity and numerical values in each cell reflected the strength and direction of the correlation coefficients, enabling an intuitive understanding of the relationships among variables.

### 2.11. Western Blot Verification

Five hundred microliters of RIPA lysis buffer (20–188, Sigma) were added to the horse and donkey seminal plasma samples, which were subsequently incubated at 4 °C overnight. The next day, each sample was ultrasonicated for 1.5 min at 15 s intervals at 150 W, and the samples were gently resuspended. Then, the samples were centrifuged at 10,000× *g* for 30 min, and the supernatant was recovered and transferred to new microcentrifuge tubes. Protein quantitative analysis was performed via spectrophotometry. A standard curve was drawn by linking the intensity of the color with the calibrator. The color intensity at 750 nm was measured using a microplate reader. One hundred micrograms of horse and donkey seminal plasma proteins were suspended in 2× loading buffer (Thermo Fisher Scientific) and loaded onto a 5–12% SDS polyacrylamide gel. The proteins were transferred to a nitrocellulose membrane at 100 V for 1.5 h. The membrane was blocked with 5% skim milk in TBS-Tween solution for 1 h at room temperature. The membrane was incubated overnight at 4 °C in blocking solution containing primary antibodies against GPX5 (1:500; 18731-1-AP, Proteintech, Rosemont, IL, USA), SERPINE2 (1:500; 66203-1-Ig, Proteintech), FOLR2 (1:500; 31264-1-AP, Proteintech), ACACA (1:500; 21923-1-AP, Proteintech), and tubulin (1:1000; ab6046, Abcam, Cambridge, MA, USA). The following day, the membrane was washed three times for 10 min each with TBS-Tween before being incubated with secondary antibody diluted at a ratio of 1:5000 for 1 h at room temperature. The membrane was subsequently washed three times for 10 min each with TBS-Tween. The protein bands were visualized using an enhanced chemiluminescence (ECL) Plus system (GEL) and observed with a Gel Doc XR+ system (Bio-Rad, Hercules, CA, USA). The bands were quantified using ImageJ (version 1.54j). The original Western blotting image is shown in Figure S1, and the target protein-to-reference ratio is in Table S1.

### 2.12. Statistical Analysis

The data were analyzed and visualized via GraphPad Prism 9.0 software (GraphPad Prism, La Jolla, San Diego, CA, USA), and one-way ANOVA and Student’s *t*-test were used for validation. Specifically, one-way ANOVA was primarily used to evaluate the differences in relevant indicators between horses and donkeys. By analyzing the ratio of between-group variance to within-group variance, it was determined whether species, as a single factor, significantly influenced each indicator. When significant differences were identified through ANOVA, pairwise comparisons were conducted using Student’s *t*-test to further examine specific differences in these indicators between horses and donkeys. The combined application of these two statistical methods allowed for a comprehensive and precise elucidation of the differences and relationships among various indicators across experimental groups. The results are presented as the means ± standard deviations (SDs), and the significance level was set at *p* < 0.05. All the experiments were conducted with three biological and technical replicates.

## 3. Results

### 3.1. Evaluation of Horse and Donkey Sperm Quality and Gestation

**Figure 2:**
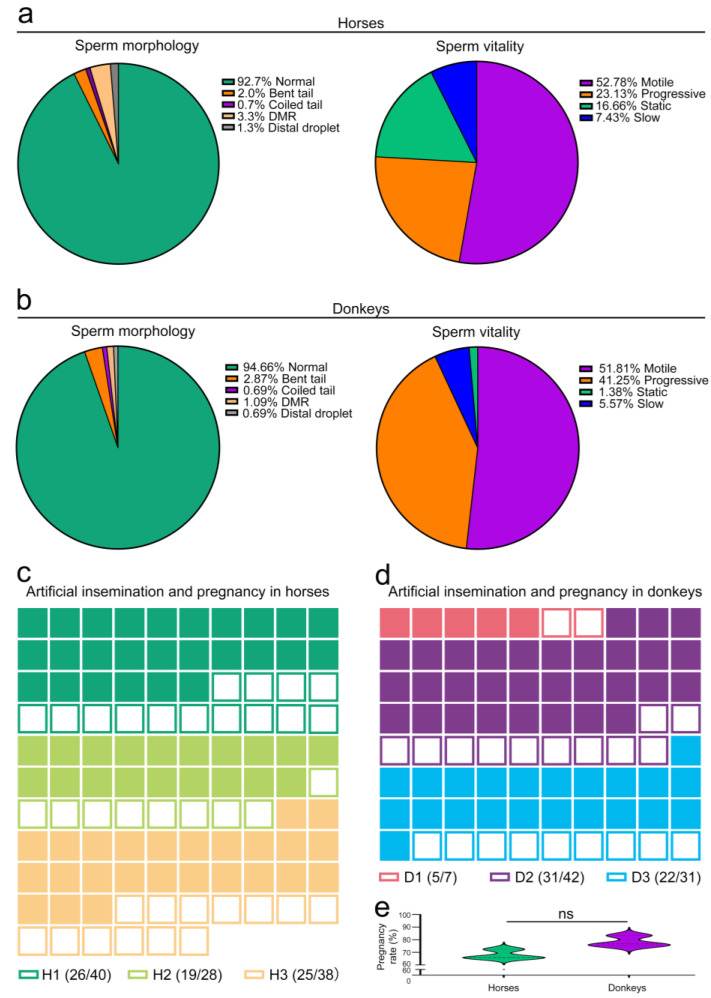
Evaluation of horse and donkey sperm quality and gestation. (**a**) CASA features of horse sperm. (**b**) CASA features of donkey sperm. (**c**) Pregnancy outcomes of artificial insemination in horses. Different colors correspond to different horses, with solid squares denoting horses that conceived after artificial insemination and hollow squares denoting horses that did not conceive. (**d**) Pregnancy outcomes of artificial insemination in donkeys. Different colors correspond to different donkeys, with solid squares denoting donkeys that conceived after artificial insemination, whereas hollow squares denote donkeys that did not conceive. (**e**) Pregnancy rates of horses and donkeys. The data are expressed as the means ± SDs. *p* < 0.05 indicates a statistically significant difference. “ns” represents not significant. H1: Horse1, H2: Horse2, H3: Horse3; D1: Donkey1, D2: Donkey2, D3: Donkey3.

To evaluate the reproductive potential of horses and donkeys, we initially analyzed the morphological and vitality characteristics of their sperm. The findings revealed that 92.7% of the horse sperm had normal morphology, with 7.3% displaying abnormalities such as bent tails and coiled tails. Furthermore, in the assessment of sperm viability, 75.9% of the sperm were motile and progressive, whereas 24.1% were slow and static (Figure 2a). In the donkey semen, 94.7% of the sperm exhibited a normal morphology, whereas 5.3% presented an abnormal morphology. A total of 93.1% of the sperm demonstrated motility and progression, with the remaining 6.9% exhibiting slow and static behavior (Figure 2b). On the basis of the above findings, the normal sperm morphology of horses and donkeys surpassed 90%, with vitality exceeding 75%, thereby satisfying the fundamental criteria for clinical artificial insemination. Subsequently, artificial insemination was conducted using test horses and donkeys. The study findings revealed that 102 mares underwent artificial insemination by the three stallions, resulting in a successful pregnancy for 69 of them and yielding a pregnancy rate of 67.6% (Figure 2c,e). Moreover, three male donkeys were utilized for the artificial insemination of 76 female donkeys, resulting in 53 pregnant female donkeys and a pregnancy rate of 76.3% (Figure 2d,e). The pregnancy success rates for both species exceeded 65%, which is a range that is consistent with normal parameters. These results led to the conclusion that the sperm morphology, vitality, and pregnancy outcomes in the horses and donkeys involved in this study met the normal situation.

### 3.2. Display of the Seminal Plasma Proteome in Horses and Donkeys

**Figure 3:**
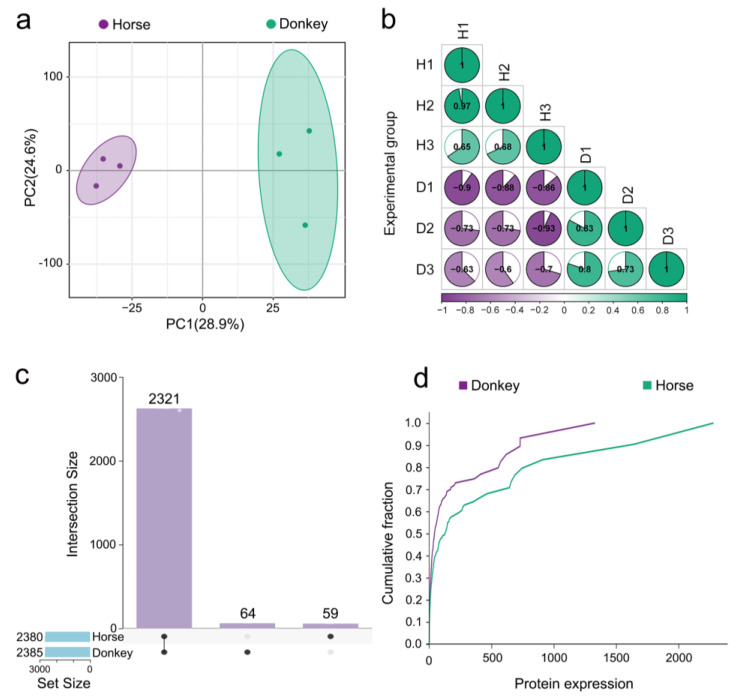
Seminal plasma proteome of horses and donkeys. (**a**) PCA of seminal plasma proteomes between horses and donkeys. (**b**) Correlation circle diagram of seminal plasma proteomes between horses and donkeys. (**c**) UpSet plot of seminal plasma between horses and donkeys. (**d**) CDC of seminal plasma proteomes between horses and donkeys.

This study employed advanced proteomic technology known as 4D-DIA to analyze both protein composition and expression levels within seminal plasma samples from horses and donkeys. Principal component analysis (PCA) revealed distinctive distribution patterns between the seminal proteomes of these two species, indicating that discernible differences exist in their respective compositions as well as their overall expression profiles (Figure 3a). Furthermore, examination via a correlation matrix revealed substantial intergroup disparities in protein expression between horse and donkey seminal plasma samples; however, strong intragroup consistency persisted (Figure 3b). Notably, an UpSet plot revealed coexpressed proteins (CEPs) between horse and donkey seminal plasma, where the total count reached 2321 proteins; additionally, specifically expressed proteins (SEPs) unique to either species were highlighted, with donkeys expressing 64 SEPs and horses exhibiting 59 SEPs (Figure 3c). The cumulative distribution curve (CDC) illustrates the frequency of protein expression distribution in different intervals of horse and donkey seminal plasma. In the 0–1400 interval, the purple line surpasses the green line, indicating higher protein expression in donkey seminal plasma within this range, with peak protein expression at 1400. Within the 1400–2300 interval, only the green line is evident, indicating greater protein expression in horse seminal plasma within this range, reaching up to 2300 (Figure 3d). These findings demonstrate distinct characteristics in protein expression between horse and donkey seminal plasma.

### 3.3. Presentation of Seminal Plasma CEPs in Horses and Donkeys

**Figure 4:**
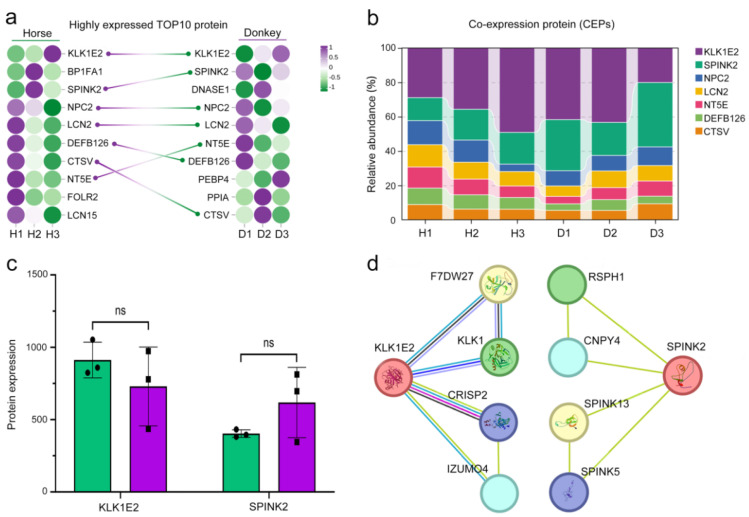
Presentation of seminal plasma CEPs in horses and donkeys. (**a**) The highly expressed TOP10 protein in the seminal plasma of horses and donkeys connects the same proteins on both sides in a straight line. Left: horse; right: donkey. (**b**) The relative abundance of 7 CEPs in the seminal plasma of horses and donkeys. (**c**) KLK1E2 and SPINK2 protein expression in the seminal plasma of horses and donkeys was determined via proteomic sequencing data. (**d**) PPIs of KLK1E2 and SPINK2 in Equus species. The data are expressed as the means ± SDs. “ns” represents not significant. H1: Horse1, H2: Horse2, H3: Horse3; D1: Donkey1, D2: Donkey2, D3: Donkey3.

To identify the characteristics of seminal plasma proteins in horses and donkeys, we conducted a comprehensive analysis of seminal plasma protein sequencing data from both species. The results revealed that the top 10 highly expressed proteins in horse seminal plasma were KLK1E2, BP1FA1, SPINK2, NPC2, LCN2, DEFB126, CTSV, NT5E, FOLR2, and LCN15. In donkey seminal plasma, the top 10 highly expressed proteins were KLK1E2, SPINK2, DNASE1, NPC2, LCN2, NT5E, DEFB126, PEBP4, PPIA, and CTSV. Notably, seven proteins were found to be present in both horse and donkey seminal plasma (Figure 4a). These CEPs include KLK1E2, SPINK2, NPC2, LCN2, NT5E, DEFB126, and CTSV. Among these proteins, KLK1E12 and SPINK2 presented the highest expression levels, with relative abundances exceeding 50% of the total abundance of these seven proteins (Figure 4b). The protein sequencing data revealed no statistically significant difference (*p* > 0.05) in the expression levels of KLK1E2 and SPINK2 between horse and donkey seminal plasma (Figure 4c). A network diagram depicting protein interactions highlighted associations with sperm quality for interacting proteins linked to KLK1E2 and SPINK2 (Figure 4d). The evidence presented above suggests that the majority of proteins identified in horse and donkey seminal plasma are likely to be identical and exhibit similar functionalities.

### 3.4. Classification of Seminal Plasma SEPs in Horses and Donkeys

**Figure 5:**
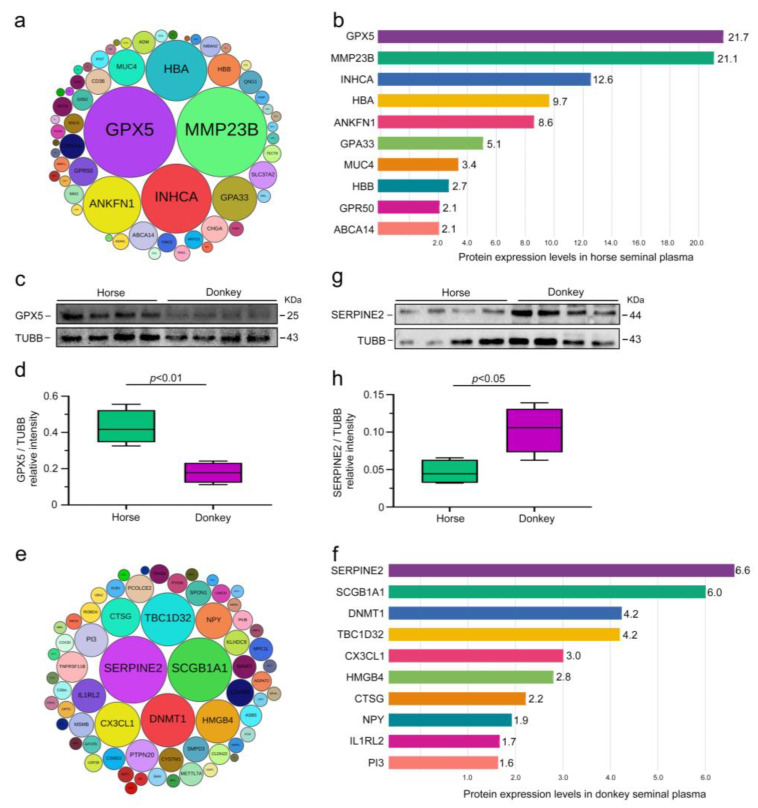
Classification of seminal plasma SEPs in horses and donkeys. (**a**) A total of 59 SEPs in horse seminal plasma, with the different-sized circles indicating the expression levels of proteins. (**b**) The expression levels of the top 10 SEPs in horse seminal plasma. (**c**) GPX5 protein expression in horse and donkey seminal plasma was determined by Western blotting. (**d**) Quantification of the relative intensity of GPX5/TUBB. (**e**) A total of 64 SEPs in donkey seminal plasma, with the different-sized circles indicating the expression levels of proteins. (**f**) Expression levels of the top 10 SEPs in donkey seminal plasma. (**g**) SERPINE2 protein expression in horse and donkey seminal plasma was determined by Western blotting. (**h**) Quantification of the relative intensity of SERPINE2/TUBB. TUBB protein expression was employed as an internal reference for normalization. *p* < 0.05 indicates a statistically significant difference.

After identifying CEPs in horse and donkey seminal plasma, our focus shifted to exploring SEPs in these samples. Given the distinct species-specific seminal plasma protein expression profiles of horses and donkeys, we identified 59 SEPs in horse seminal plasma (Figure 5a). Notably, the top 10 proteins included GPX5, MMP23B, INHCA, HBA, ANKFN1, GPA33, MUC4, HBB, GPR50, and ABCA14 (Figure 5b). Western blot analysis was employed to validate the accuracy of the proteomic sequencing results and identify SEPs in horse seminal plasma. The findings revealed significantly greater expression of the GPX5 protein in horse seminal plasma than in donkey seminal plasma (*p* < 0.05) (Figure 5c,d). We subsequently identified 64 SEPs in donkey seminal plasma (Figure 5e), with SERPINE2 being one of the top 10 proteins, along with SCGB1A1, DNMT1, TBC1D32, CX3CL1, HMGB4, CTSG, NPY, ILIRL2, and PI3 (Figure 5f). Similarly, Western blot analysis for validation confirmed markedly higher expression of the SERPINE2 protein in donkey seminal plasma than in horse seminal plasma (*p* < 0.05) (Figure 5g,h). These experimental results were consistent with the sequencing data, indicating distinct protein expression profiles between horse and donkey seminal plasma.

### 3.5. Comparison of Seminal Plasma DEPs in Horses and Donkeys

**Figure 6:**
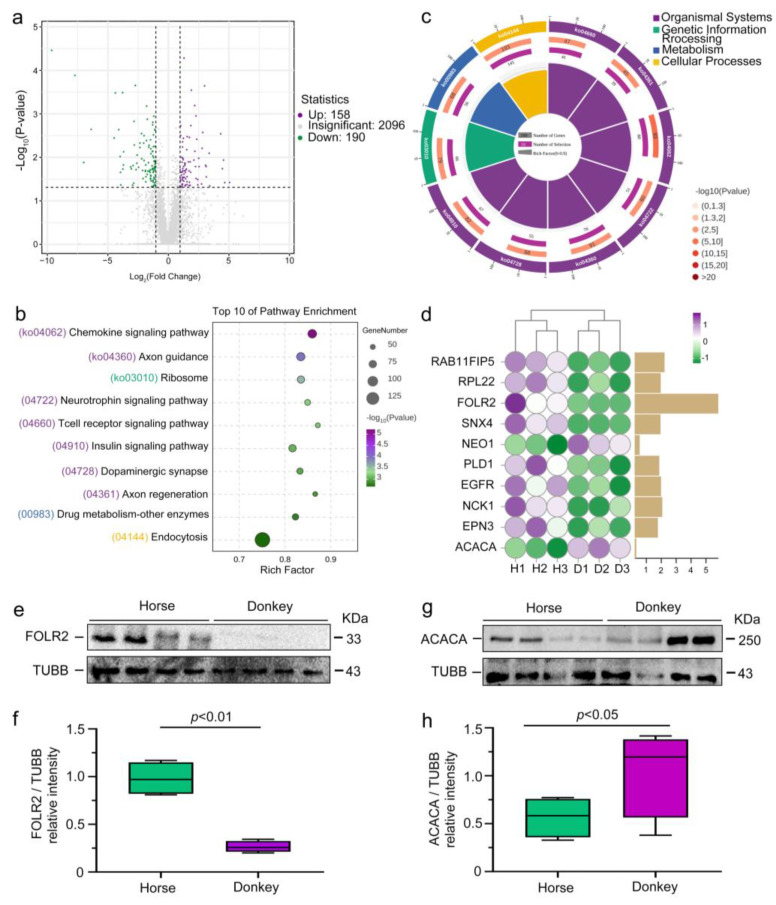
Comparison of seminal plasma DEPs in horses and donkeys. (**a**) Volcano plot of horse and donkey seminal plasma DEPs, with downregulated and upregulated proteins represented by green and purple dots, respectively. (**b**) Enrichment circle diagram displaying the primary classification of the top 10 KEGG signaling pathways in horse and donkey seminal plasma. (**c**) Bubble chart displaying the three-level classification of the top 10 KEGG signaling pathways in horse and donkey seminal plasma. (**d**) The expression of the top 10 DEPs in horse and donkey seminal plasma. (**e**) FOLR2 protein expression in horse and donkey seminal plasma was determined by Western blotting. (**f**) Quantification of the relative intensity of FOLR2/TUBB. (**g**) ACACA protein expression in horse and donkey seminal plasma was determined by Western blotting. (**h**) Quantification of the relative intensity of ACACA/TUBB. TUBB protein expression was employed as an internal reference for normalization. *p* < 0.05 indicates a statistically significant difference. H1: Horse1, H2: Horse2, H3: Horse3; D1: Donkey1, D2: Donkey2, D3: Donkey3.

Following comprehensive proteomic data analysis, we identified 158 upregulated proteins (purple dots) and 190 downregulated proteins (green dots) in donkey seminal plasma compared with horse seminal plasma. In comparison, 2096 proteins were not significantly different between horse and donkey seminal plasma (gray dots) (Figure 6a). We conducted further analysis of the DEPs via the KEGG database. The results of the KEGG primary classification revealed that the DEPs were predominantly enriched in organismal systems, genetic information processing, metabolism, and cellular processes (Figure 6b). After conducting a more comprehensive classification, we identified and presented the top 10 signaling pathways, including the chemokine, axon guidance, ribosome, neurotrophin, T-cell receptor, insulin, dopaminergic synapse, axon regeneration, drug metabolism, other enzymes, and endocytosis signaling pathways, on the basis of the third-level KEGG classification (Figure 6c). These top 10 KEGG signaling pathways were associated with numerous DEPs. Among them, the top 10 DEPs with the highest expression levels included RAB11FIP5, RPL22, FOLR2, SNX4, NEO1, PLD1, EGFR, NCK1, EPN3, and ACACA (Figure 6d). After analyzing the top 10 DEPs in horse and donkey seminal plasma, we detected higher expression levels of most proteins in horse seminal plasma than in donkey seminal plasma, with the exception of the NEO1 and ACACA proteins. Notably, FOLR2 expression was significantly elevated. To validate the proteomic findings, we conducted Western blot experiments targeting the FOLR2 and ACACA proteins identified from the horse and donkey seminal plasma proteomes. The results demonstrated markedly increased expression of the FOLR2 protein in horse seminal plasma relative to that in donkey seminal plasma (Figure 6e,f). Conversely, the expression of the ACACA protein was notably lower in horse seminal plasma than in donkey seminal plasma (Figure 6g,h). These findings align with the outcomes of 4D-DIA proteomics, validating the precision of the analysis.

### 3.6. Correlation Between Horse and Donkey Seminal Plasma Protein and Sperm Quality

**Figure 7:**
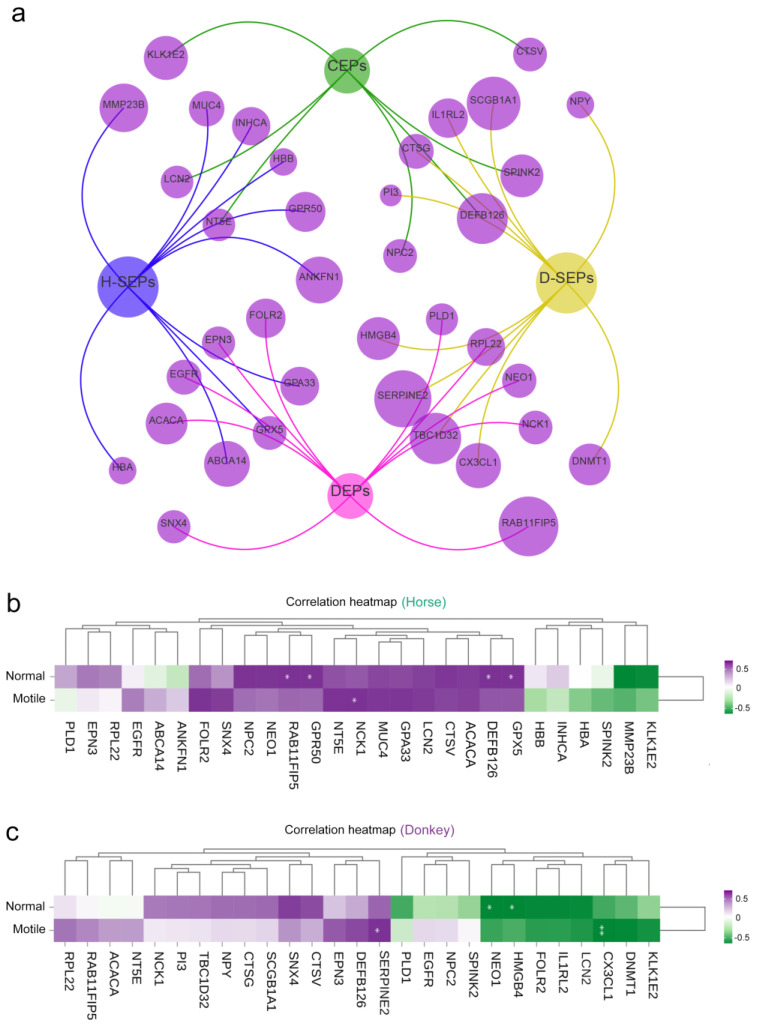
Correlation between horse and donkey seminal plasma protein and sperm quality. (**a**) The PPI network of horse and donkey seminal plasma CEPs, SEPs, and DEPs. H-SEPs: horse seminal plasma SEPs. D-SEPs: donkey seminal plasma SEPs. (**b**) The correlation between horse seminal plasma protein and normal sperm morphology and motility. (**c**) The correlation between donkey seminal plasma protein and normal sperm morphology and motility. The scale (0.5 to −0.5) in color indicates whether the correlation is positive (purple) or negative (green). A single asterisk (*) indicates a statistical difference (*p* < 0.05); a double asterisk (**) indicates a significant statistical difference (*p* < 0.01).

The protein characteristics in the seminal plasma of horses and donkeys that were identified and analyzed can provide more accurate and reliable information for assessing the reproductive potential of equine animals. On the basis of these findings, we constructed a PPI network using seminal plasma proteins (CEPs, SEPs, and DEPs) in horses and donkeys (Figure 7a). We assessed the relationships between CEPs, SEPs, and DEPs in horse and donkey seminal plasma and sperm with normal morphology and motility. The correlation matrix revealed that normal morphology indicators of horse sperm were positively correlated with the GPX5, ACACA, GPR50, and RAB11FIP5 proteins in horse seminal plasma (*p* < 0.05). Additionally, the motility indicators of horse sperm were positively correlated with the NCK1 protein in horse seminal plasma (*p* < 0.05) (Figure 7b). On the other hand, the normal morphology indicators of donkey sperm were negatively correlated with the HMGB4 and NEO1 proteins in donkey seminal plasma (*p* < 0.05). The motility indicators of donkey sperm were positively correlated with SERPINE2 proteins in donkey seminal plasma, whereas the CX3CL1 protein was negatively correlated with donkey sperm motility (*p* < 0.05) (Figure 7c). Surprisingly, the GPX5 and SERPINE2 proteins were the SEPs in horse and donkey seminal plasma, respectively. Hence, we are even more confident that the detection of GPX5 and SERPINE2 in the seminal plasma of horses and donkeys serves as a reliable indicator for evaluating sperm quality and reproductive potential in equids.

## 4. Discussion

The comparative analysis of proteomic characteristics in the seminal plasma of horses and donkeys provides novel insights into the complex molecular mechanisms regulating reproductive biology in these equine species. Through systematic profiling and comparison of the protein composition in seminal plasma, this study has identified key differences that likely account for the distinct reproductive traits and fertility outcomes observed between horses and donkeys. These variations in protein profiles not only highlight the evolutionary divergence of these species but also shed light on the molecular adaptations essential for optimal sperm function. Gaining a deeper understanding of these proteomic distinctions is critical, as it could help bridge the knowledge gap in equine reproductive physiology and inform the development of more effective breeding strategies and conservation efforts tailored to the unique biological requirements of horses and donkeys.

With regard to sperm quality, the normal morphology of horse sperm in this study was 92.7%, and the vitality (motile and progressive) was 75.9%. These values exceeded those reported for fertile stallions in a previous study on seasonal variations in sperm characteristics of fertile and infertile horses, where the normal morphology of sperm in fertile stallions was 67.1%, and the vitality was 68.2% [21]. The normal morphology of donkey sperm was also measured at 94.7%, with a vitality (motile and progressive) rate of 93.1%. These results are closely aligned with those reported in previous studies, which documented a normal morphology rate of 92.2% and a vitality rate of 76.0% in their analysis of donkey sperm morphology [22]. Consequently, the horse and donkey sperm utilized in this research met established standards for breeding. Seminal plasma protein, an essential constituent of semen, strongly influences sperm quality [23]. In this investigation, sperm-quality-qualified horses and donkeys were utilized for artificial insemination. The findings revealed a pregnancy rate of 67.6% in horses, closely aligning with the 66.7% rate. In contrast, the pregnancy rate in donkeys was notably higher at 76.3%, which strongly contrasts with the previously reported rate of 27.1% [4]. However, previous studies have reported a pregnancy rate as high as 70% in donkeys [24]. These data differ, but when animals with high-quality sperm mate, their pregnancy success rate is influenced by various factors, including breeding techniques and environmental factors. Certainly, the female factor plays a crucial role in determining the pregnancy rate, indicating that it is not exclusively dependent on the male side [25]. Nevertheless, the detection of seminal plasma protein remains an important approach for evaluating sperm quality and predicting the success rate of pregnancy.

In this study, 4D-DIA was used to generate the latest protein profiles of horse and donkey seminal plasma. We identified 2380 proteins in horse seminal plasma and 2385 in donkey seminal plasma. These figures differ slightly from the protein quantities previously reported by researchers for horse and donkey seminal plasma. Previously, some scientists utilized a combination of tandem mass tag (TMT) peptide labeling and the LC-MS/MS method to identify 1702 proteins in horse seminal plasma and 866 proteins in donkey seminal plasma [26,27]. Both of these numbers are lower than the number of proteins sequenced in our study. This disparity may be attributed to methodological distinctions, as more advanced 4D-DIA technology can discern subtly expressed proteins in horse and donkey seminal plasma.

We observed that 7 of the top 10 highly expressed proteins in the seminal plasma of horses and donkeys were identical. They are KLK1E2, SPINK2, NPC2, LCN2, NT5E, DEFB126, and CTSV. This finding suggests that there might be similarities in the protein components of the seminal plasma of horses and donkeys. Moreover, these proteins are closely associated with sperm quality and pregnancy. For example, researchers have demonstrated that the KLK1E2 protein is utilized to predict the overall pregnancy rate related to the ejaculate volume of stallions [28]. Additionally, the SPINK2 protein in seminal plasma was identified as a crucial factor for avian fertility. This conclusion was supported by the discovery that the concentration of SPINK2 in seminal plasma is positively correlated with male fertility in chicken lines exhibiting highly contrasting genetic backgrounds (broiler and layer lines) [29]. Mice with a knockout of the NPC2 protein have sperm with a deficient cholesterol content and low in vitro fertilization capacity. Hence, NPC2 is positively correlated with the fertility of male mice [30]. Furthermore, LCN2 can bind to membrane phosphatidylethanolamine, enhance lipid raft reorganization through a PKA-dependent mechanism, and promote the acquisition of fertility in sperm by facilitating cholesterol efflux [31]. Previous studies have reported that NT5E may combine with other genetic markers to increase the genetic ability of cattle [32]. A glycosylated polypeptide, β-defensin 126 (DEFB126), originates from the epididymis and can adsorb on the surface of sperm [33]. Studies have indicated that the proportion of DEFB126 in the sperm of macaques and humans is positively correlated with sperm motility and normal morphology [34,35]. CTSV can act as a key regulatory protein for blastocyst development [36]. These proteins are directly associated with the reproductive capacity of animals. As a result, the CEPs identified in horse and donkey seminal plasma may also play a crucial role in assessing the reproductive potential of the equine family.

The SEPs in the seminal plasma of horses and donkeys can also become potential elements for predicting the fertility of their respective species. In this study, we discovered 59 SEPs in the seminal plasma of horses, with the top 10 being GPX5, MMP23B, INHCA, HBA, ANKFN1, GPA33, MUC4, HBB, GPR50, and ABCA14. We paid particular attention to the GPX5 protein, not only because its expression was the highest in the seminal plasma of horses but also because studies have demonstrated that GPX5, an antioxidant enzyme that protects the sperm membrane from oxidative damage and is a biomarker of the fertility of boars, is often positively correlated with the boar fertility index and farrowing rate [37]. However, researchers put forward the opposite view that the GPX5 protein content is negatively correlated with sperm membrane integrity and total sperm motility [38]. So we initially identified GPX5 as a potential predictor of horse reproduction. In the seminal plasma of donkeys, we identified a total of 64 different proteins, and the top 10 proteins with the highest expression were SERPINE2, SCGB1A1, DNMT1, TBC1D32, CX3CL1, HMGB4, CTSG, NPY, ILIRL2, and PI3. The expression of the SERPINE2 protein in the seminal plasma of donkeys was the highest among the top 10 SEPs. SERPINE2 is a potent serine protease inhibitor that regulates the activity of plasminogen activators and thrombin, is related to many biological processes, and is expressed mainly in the seminal vesicle [39]. Previous studies have reported that SERPINE2 can prevent and reverse sperm capacitation in vitro, prevent sperm from binding to oocytes, and thus prevent fertilization [39,40]. However, the role of SERPINE2 in seminal plasma has not been reported. Therefore, we speculate that the role of SERPINE2 in seminal plasma is completely different from that in sperm and that its specific high expression in the seminal plasma of breeding donkeys may be a potential factor for predicting donkey reproduction.

The comprehensive proteomic analysis presented in this study provides valuable insights into the differences between horse and donkey seminal plasma proteomes. The identification of 158 upregulated and 190 downregulated proteins in donkey seminal plasma compared with horse seminal plasma highlights the existence of species-specific proteomic signatures. This observation is particularly important for understanding the unique reproductive characteristics of these two species. The KEGG analysis revealed the involvement of DEPs in organismal systems, genetic information processing, metabolism, and cellular processes. The identification of the top 10 signaling pathways associated with the DEPs provides a deeper understanding of the potential functional roles of these proteins [41,42,43]. The presence of these pathways in the seminal plasma proteome suggests their potential involvement in regulating reproductive functions [44]. Notably, most of the top 10 DEPs with the highest expression levels were more abundant in horse seminal plasma than in donkey seminal plasma. This finding suggests that species-specific differences in the expression levels of these proteins may exist, which could contribute to the distinct reproductive traits observed in horses and donkeys. The significantly elevated expression of FOLR2 in horse seminal plasma is particularly interesting, as this protein has been implicated in various biological processes [45,46]. ACACA is the key enzyme that controls the rate of fatty acid synthesis to regulate lipid metabolism [47]. The relatively high expression of this protein in donkey seminal plasma may suggest a species-specific role in regulating reproductive functions. The validation of the proteomic findings through Western blot experiments targeting the FOLR2 and ACACA proteins further strengthens the credibility of this study. The markedly increased expression of the FOLR2 protein in horse seminal plasma and the notably lower expression of the ACACA protein in horse seminal plasma than in donkey seminal plasma align with the proteomic results. This validation confirms the accuracy of the 4D-DIA proteomic analysis and reveals DEPs in the seminal plasma of horses and donkeys.

**Figure 8:**
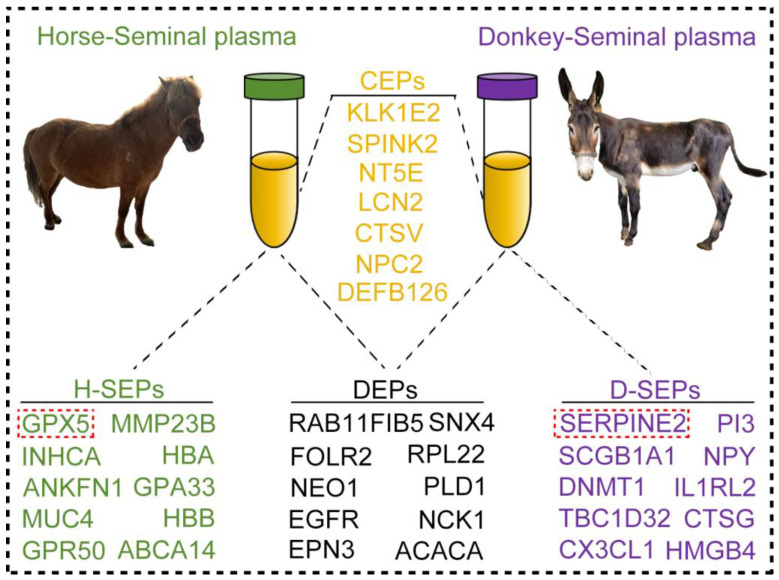
Protein characteristics in horse and donkey seminal plasma. The orange text represents CEPs in horse and donkey seminal plasma, the black text represents DEPs in horse and donkey seminal plasma, the green text represents SEPs in horse seminal plasma, and the purple text represents SEPs in donkey seminal plasma. The red frame highlights the identification of protein markers in the seminal plasma of horses and donkeys, as revealed by this study.

The normal morphology of sperm constitutes the foundation of its functional integrity, and capacitation represents an essential physiological process that sperm must undergo prior to fertilization [48]. These proteins within seminal plasma guarantee that sperm can acquire fertilization ability at the opportune time point by regulating the stability of the sperm cell membrane and facilitating intracellular signal transduction [49,50]. On the basis of the CEPs, SEPs, and DEPs we previously discovered, Pearson correlation analysis revealed that the GPX5 protein in horse seminal plasma positively correlates with the normal morphology of horse sperm. Moreover, the SERPINE2 protein in donkey seminal plasma is positively associated with sperm motility. Coincidentally, the GPX5 and SERPINE2 proteins in horse and donkey seminal plasma are the SEPs with the highest expression levels. These findings suggest that the GPX5 and SERPINE2 proteins may serve as potential indicators of superior sperm quality in horses and donkeys. Although there are numerous similarities in the types and functions of seminal plasma proteins between horses and donkeys, certain disparities in protein composition and content between horses and donkeys still exist. These differences might reflect the variations in reproductive strategies and adaptability to the reproductive environment among different species [51]. Hence, the seminal plasma proteins mentioned in this study may serve as key factors for analyzing the characteristics of horses and donkeys (Figure 8).

In the comparative analysis of horse and donkey seminal plasma, differences in seminal plasma protein were observed, reflecting variations in reproductive physiology between the two species. These disparities are evident in the composition and levels of seminal plasma proteins. These findings offer valuable insights into the reproductive characteristics of horses and donkeys at a relatively deep level. However, this study has certain limitations. The sample size was relatively small, which may affect the statistical power and generalizability of the results. Additionally, further functional validation of the identified proteins is needed to fully elucidate their roles in reproductive processes. Future research could incorporate larger sample sizes, include different breeds and populations, and utilize advanced proteomic techniques to gain a more in-depth understanding of the proteomic landscape of equine seminal plasma.

## 5. Conclusions

In conclusion, this study offers a thorough comparative analysis of the proteomic characteristics in seminal plasma between horses and donkeys, revealing distinct differences in protein profiles that are likely linked to their reproductive biology and species-specific physiological functions. Overall, this research constitutes a substantial advancement in the field of comparative reproductive proteomics. The insights derived from this study not only pave the way for exploring the molecular basis of reproductive differences between horses and donkeys but also hold promise for guiding the development of innovative strategies to enhance equine reproduction and fertility.

## Data Availability

All data are obtainable via the article and its Supplementary Materials file, or upon request addressed to the authors.

## Supplementary Materials

The following supporting information can be downloaded at: https://www.mdpi.com/article/10.3390/ani15111532/s1. Figure S1: Uncropped Western blot figures. Table S1: Gray values of Western blot.
