# Supplementary figures and images for: Comparative Analysis of Proteomic Characteristics in Seminal Plasma Between Horses and Donkeys
Source: Animals (Basel). 2025 May 23;15(11):1532. doi: 10.3390/ani15111532 (PMC12153549; doi:10.3390/ani15111532)

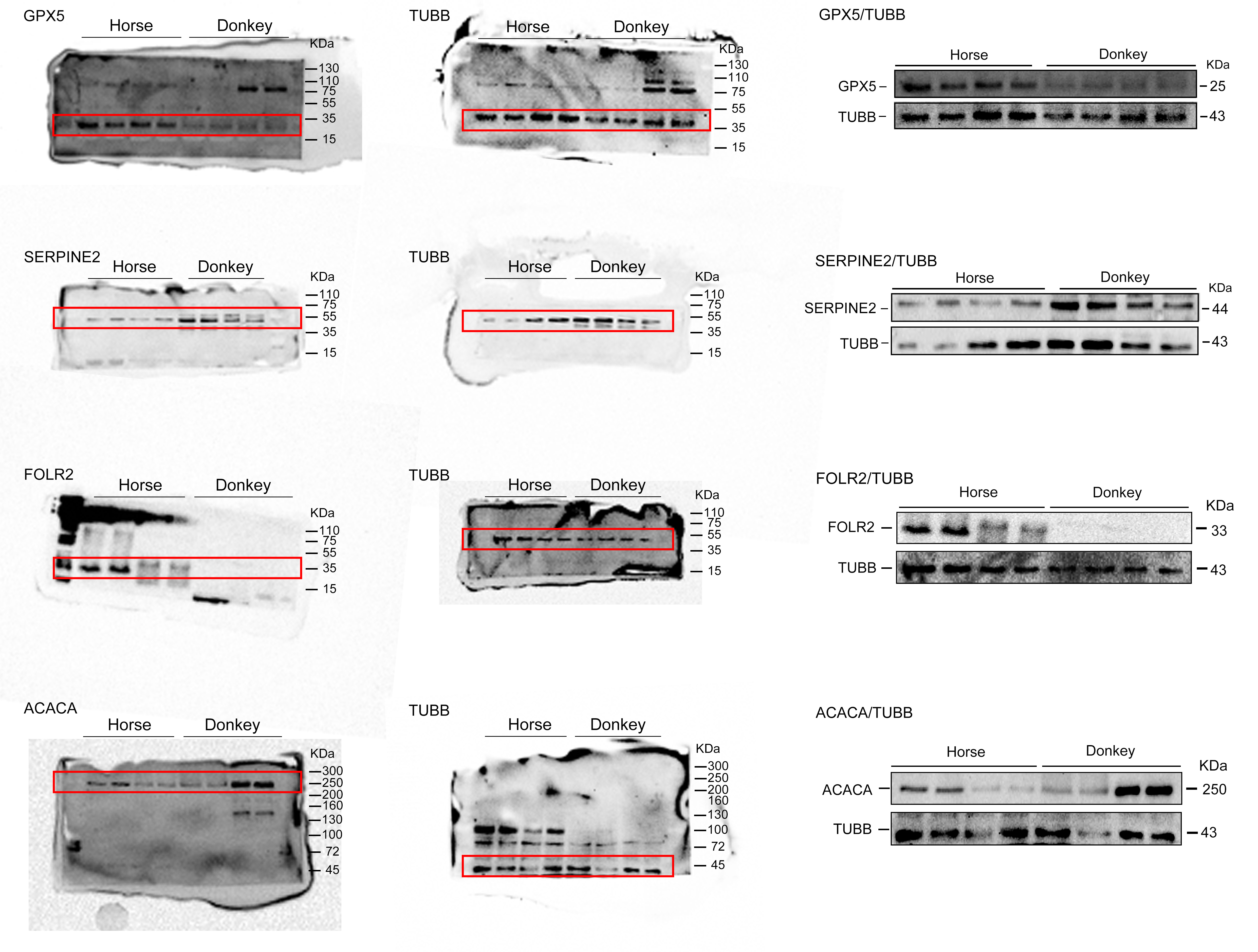

Supplement: Supplementary file 1 [file animals-15-01532-s001.zip › Figure S1. Uncropped western blot figures.jpg]
